# Supplementary material for: Microstructural Effects on Irradiation Creep of Reactor Core Materials
Source: Materials (Basel). 2023 Mar 13;16(6):2287. doi: 10.3390/ma16062287 (PMC10054019; doi:10.3390/ma16062287)
Supplement: Supplementary file 1 [file materials-16-02287-s001.zip › materials-2219805-supplementary.pdf]

Supplementary Material – Fffects of Microstructure on Irradiation Creep of Reactor Core Materials

The two main processes that are believed to be important for irradiation creep are stress-induced climb and glide (SICG) or simple mass transport. There is considerable debate over which process dominates and at what temperatures and stresses. Whereas one can say that SICG is likely to be the most dominant process at high temperatures (> 500 °C) and stresses (> 200 MPa), or at very low temperatures (< 100 °C) in austenitic alloys, there are ranges of temperatures and stresses where both dislocation glide and diffusional mass transport can be significant. There are uncertainties concerning either mechanism, but it is reasonable to assume that both depend either partly or wholly on diffusional mass transport.

An appreciation for the physical processes controlling irradiation creep, and therefore whether SICG or diffusional mass transport are the dominant strain producing mechanisms, can be obtained by reviewing the evidence for both mechanisms in the context of the physics behind each. There is a wealth of irradiation creep data for Zr-2.5Nb pressure tubes that has been accrued over many years because, unlike pressure vessel reactors, these tubes are the pressure retaining components in a CANDU reactor core. This document describes the two main theoretical mechanisms that are used to model irradiation creep (mass transport and SICG) for Zr-2.5Nb pressure tubing.

Case Study – Irradiation Creep of Zr-2.5Nb Pressure Tubing

S1. Microstructure of Zr-2.5Nb Pressure Tubing

The microstructure of Zr-2,5Nb is illustrated in Figure S1, which shows that the grain structure consists mostly of flattened platelets having minor axis dimensions ranging from 0.1 – 0.6 µm.

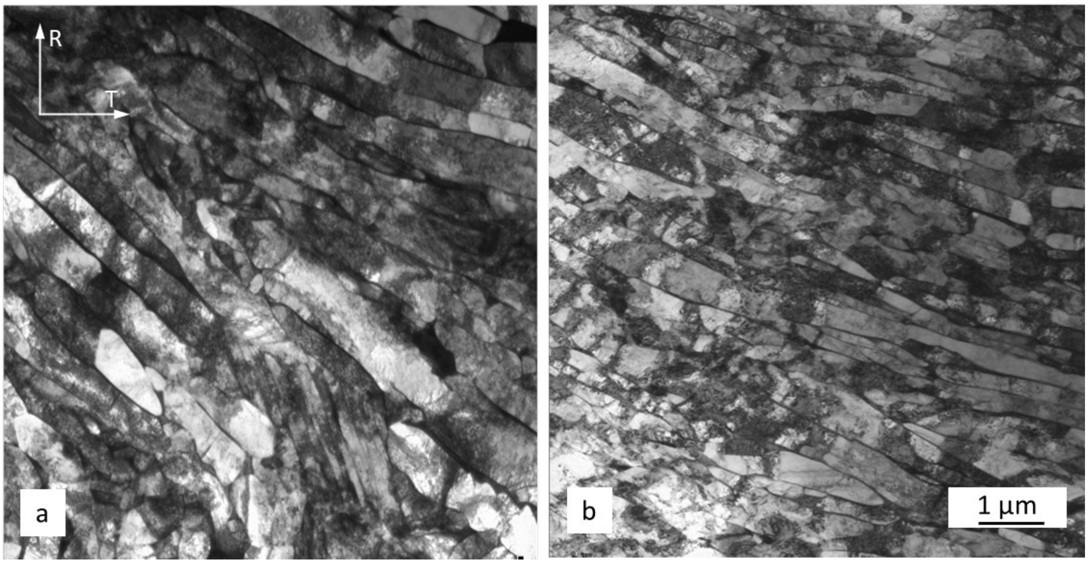

Figure S1. Examples of grain structures in a typical Zr-2.5Nb pressure tubing viewed looking down the longitudinal axis of the pressure tube for: (a) the front end and (b) the back end.

Because Zr-alloys are comprised mostly of hexagonal close-packed (HCP) grains their properties are very sensitive to the texture, i.e. the distribution of grain orientations. For different Zr-alloy components the textures are illustrated in Figure S2.

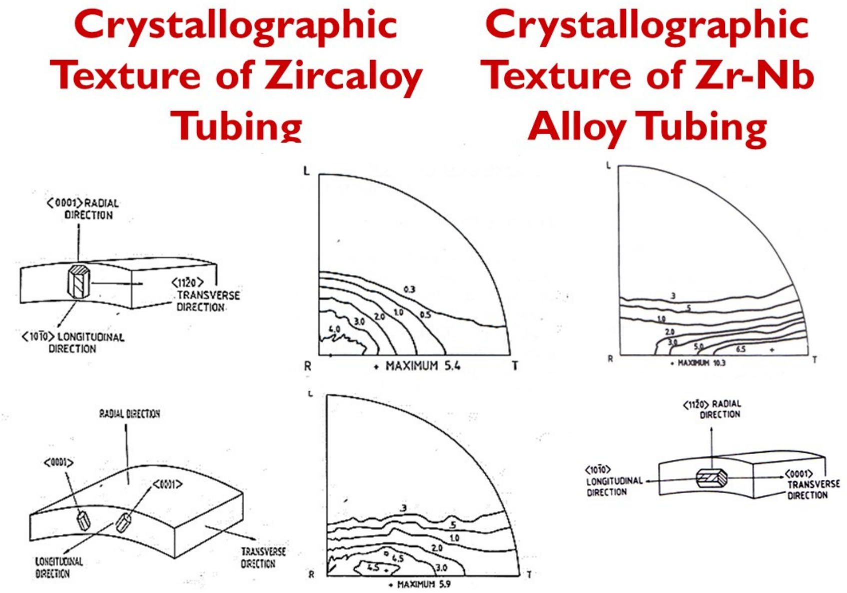

Figure S2. Basal pole figures illustrating the distribution of the unique c-axes of grains in different Zr-alloy components.

For Zr-2.5Nb pressure tubing the grain structure and the crystallographic orientations of the grains are very important for irradiation creep. The grain size and shape tend to correlate with the crystal orientation; the more equiaxed grains having their c-axes orientated toward the radial direction, Figure S3. The green and blue grains have c-axes close to the radial directions and tend to be more equiaxed when viewed looking down the longitudinal axis of the tube. Grains that are flatter (the red grains) tend have their c-axes oriented towards the transverse (hoop) direction of the tube.

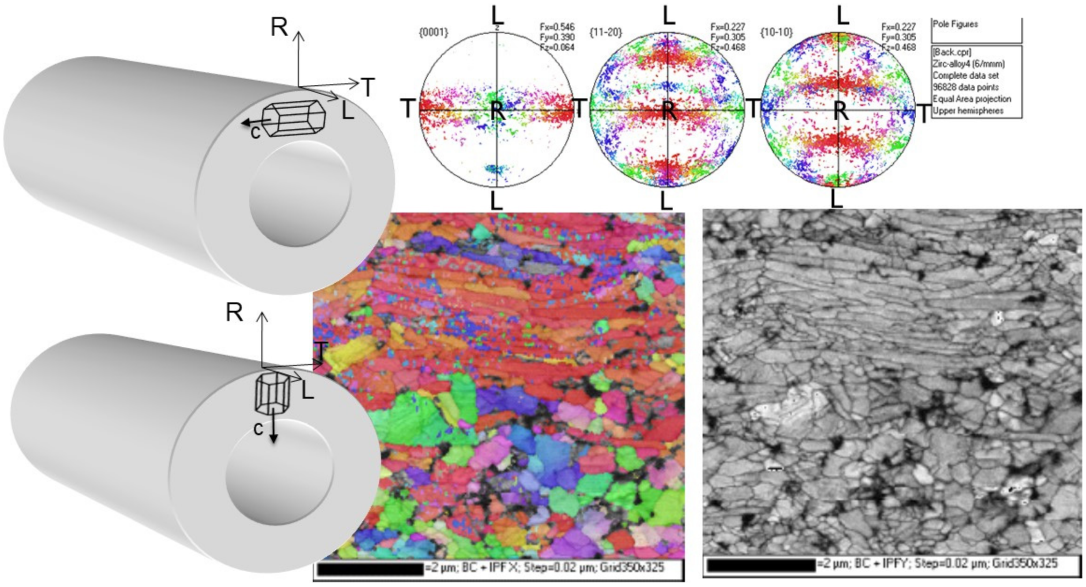

Figure S3. Transmission Kikuchi Diffraction (TKD) pole figures and image quality maps showing the relationship between grain orientations and grain shapes in a sample of Zr-2.5Nb pressure tubing. Reprinted/adapted with permission from [2]. 2019, Elsevier.

Grains are typically thinner (smaller minor axis dimension) at the end of the tube corresponding with the back-end of the extrusion, Figure S4. Also shown in Figure S4 are quarter pole figures indicating the tendency for the basal poles to be oriented more towards the radial direction of the tube at the back-end. The basal texture is represented by Kearns texture parameters [1], which are factors (f_R, f_T f_L) that resolve and sum the single crystal tensor properties (such as strain for example) corresponding with the c-axis of the single crystal onto the directions of interest in the component (R, T, L).

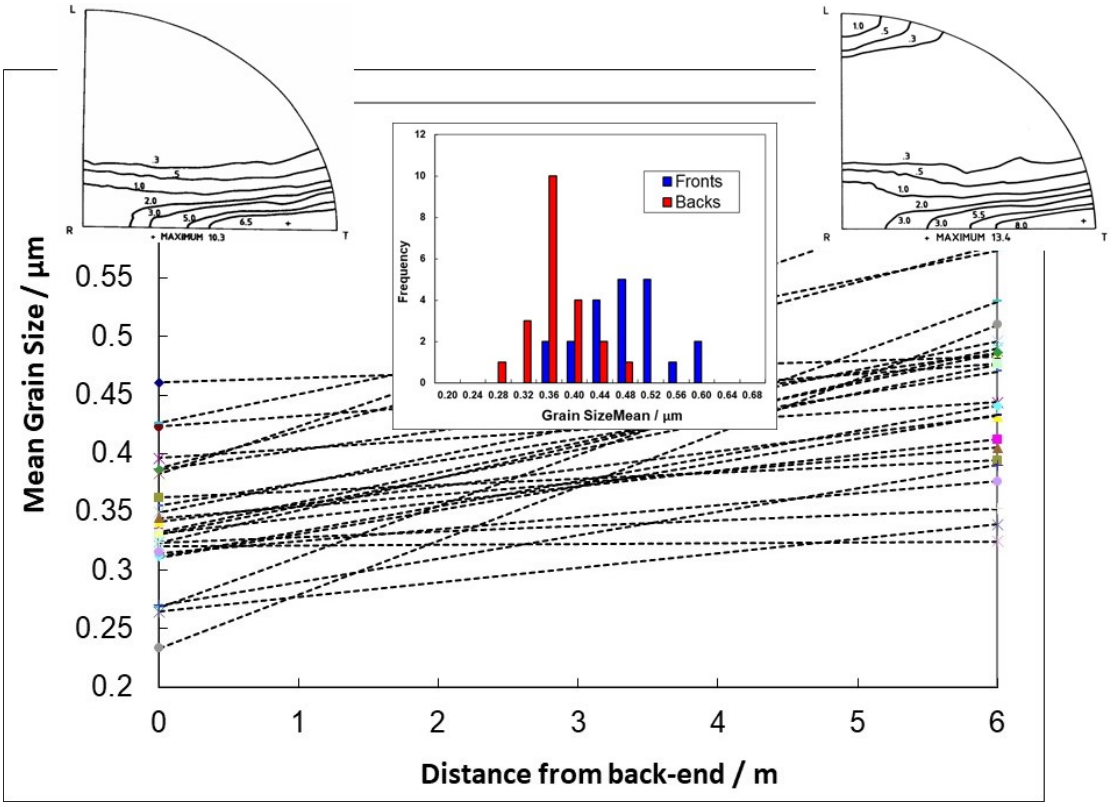

Figure S4. Microstructure data for Zr-2.5Nb pressure tubing installed in different CANDU reactors showing minor axis grain dimensions and the corresponding basal pole figures centred on the radial direction. The grain thickness is smaller, and the basal pole texture shows that the grains tend to have their unique c-axes oriented slightly more towards the radial direction at the back-end (the end leaving the extrusion press last).

Perhaps the most important observation concerning the irradiation creep of pressure tubes in CANDU reactors is the observation that some tubes (designated TG3 RT1), fabricated with

unusual microstructures, exhibited unusually high rates of diametral creep and correspondingly low rates of elongation [2, 3]. The balance between the elongation and diametral strain rates indicates that it is not the magnitude of the creep that is affected but rather the anisotropy. Given that the TG3 RT1 tubes were fabricated with a low dislocation density, because that was deemed to be a factor in producing a tube with low elongation [4], there is reason to doubt that the relatively high diametral creep could be attributed to a high dislocation density given the deviatoric stress state in the pressurised tube is approximately (-60, +60, 0) MPa in the radial, transverse and longitudinal directions (R, T, L) respectively, for a typical internally pressurised CANDU pressure tube. It could then be argued that the low dislocation density and high diametral creep are inconsistent with a SICG mechanism. However, it has been proposed that dislocation loops produced by irradiation grow could form a dislocation network [5], thus circumventing the difficulty in explaining the high diametral creep when the network dislocation density is low.

S2 Modelling Irradiation Creep by Stress Induced Climb and Glide (SICG)

Stress induced climb and glide depends principally on the glide of network dislocations introduced by cold-working during fabrication. The network dislocations produced by dislocation slip are fundamentally different from the prismatic loops produced by the clustering of point defects. Loop evolution is by climb and is therefore governed by diffusional mass transport (rate theory). There have been claims that dislocation loops evolve into an equivalent network and therefore, before one discusses the role of network dislocations in irradiation creep, it is instructive to distinguish between the two types of dislocations.

In the 2020 creep review by Onimus et al. [5] a creep mechanism was proposed whereby dislocation loops grow and form a network that can glide and create a plastic shear strain that is in addition to the dilatational strain from the loops themselves. Whereas shearing of a prismatic loop that redistributes the strain within the loop's glide cylinder is conceivable, the proposed mechanism results in the creation of additional strain that, in retrospect, is both unrealistic and unphysical. Figure 10 from Onimus et al. [5] shows the initial distortion of a prismatic loop in a small single crystal and the effect of an applied shear stress. The dislocations are allowed to glide subject to the applied stress following a Peach-Koehler rule and the resulting plastic distortion was depicted as reproduced in Figure S5. There are two problems with this scenario: (i) the net strain from the glide of the loop does not conform to work done by the applied shear stress; (ii) the elastic strain distortion observed at the boundary surface of the single crystal does not relax as the dislocation half planes move away from each surface. The areas red-circled in Figure S5 show that the planes in the original figure retain their plastic distortion even when the dislocation has moved away from the surface and left the crystal. The planes should relax once the dislocation has moved away in the direction of the red arrows. For example, at (c) there is nothing to maintain the elastic distortion of the crystal shown in the region circled and it must relax to the right. Likewise for the elastic distortion at the opposite surface in (e) that should be fully relaxed to the left once the dislocation has exited the left side of the crystal. The final adjustment shown in the original figure (shown in Figure S5(f)) is applied as a response to the shear stress. Quite apart from invoking a non-existent force to maintain the position of the planes once the dislocations have exited the crystal, there is no reason for the planes to be further

displaced in the direction depicted. This would apply the same whether one was considering a discrete prismatic loop or a large loop that had formed from the coalescence of other loops in the same plane.

If one considers a shear stress of 50 MPa (say), and a shear modulus of 35 GPa, the elastic strain is $<\frac{1}{10}$th of a degree, an imperceptible distortion relative to what is shown. The elastic distortion from the applied stress would put the equilibrium position of the planes (which would be a shear of $<\frac{1}{10}$th of a degree) in the final two frames moving inwards toward the elastic equilibrium position dictated by the imposed shear stress and not in the opposite direction as depicted. Apart from the unphysical nature of the proposed mechanism, any dislocation movement in a polycrystalline specimen would be against constraints from neighbouring grains. Even though the material in neighbouring grains will be distorted by similar small elastic strains subject to an applied stress (depending on shear modulus), the elastic displacement is small and insufficient to cause the plastic distortion that has been envisaged in (f).

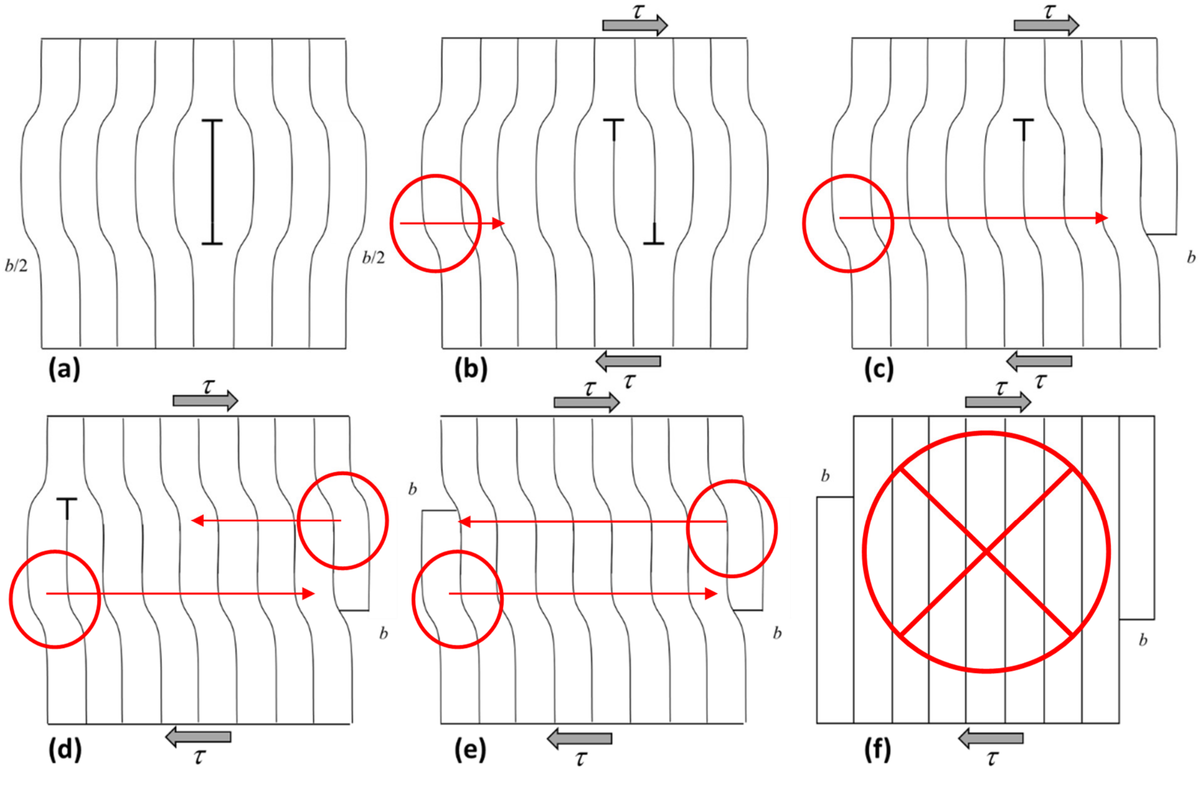

Figure S5. Depiction of creep distortion of a prismatic loop according to the rule described in [5]. The red arrows denote the direction one would expect the planes to relax given that the elastic shear strain is too small to maintain the distortion of the planes after the dislocations had exited the crystal as originally depicted. Reprinted/adapted with permission from [5]. 2019, Elsevier.

If the prismatic loops were to move, it would be by translation within their glide cylinders. These arguments are moot to some extent because there is no experimental evidence to support

the notion that dislocation loops evolve to form a network and then produce strain that is in addition to that from the climb of the prismatic loops themselves. As stated by Woo et al. [6], "At the temperatures and applied stresses at which irradiation creep is important it may not even be possible to activate these [slip] systems. This could explain why the creep rates of annealed zirconium alloys are always low [7, 8] even though a high dislocation density in the form of a-type dislocation loops evolves during irradiation."

The model of pressure tube of pressure tube deformation published by Christodoulou et al. [9, 10] is primarily based on dislocation slip of network dislocation. The model was built on the premise that the biggest driver for irradiation creep in Zr-2.5Nb pressure tubes was SICG taking into account grain interaction stresses in a polycrystalline solid. The model depended on establishing a single crystal response to any stress and from there the polycrystal response could be calculated by combining the creep responses of the individual grains. A key element was the fact that the single crystal response was anisotropic and therefore the polycrystal response would also be anisotropic unless the grains were randomly oriented. The single crystal response was described assuming that climb and glide was the strain producing mechanism but it is was also stated that the model could be generalised to any strain producing mechanism [6, 11]. The polycrystal response could be modelled by combining the strains from individual grains in a polycrystalline model.

Without considering grain interactions, if each grain is allowed to deform according to the activated slip systems and, when the resulting strain is added to the total, the resulting summation would give a so-called lower-bound result. However, the model described by Christodoulou et al. [9, 10] adopted the more realistic approach by allowing for grain-to-grain incompatibility. This was achieved by allowing each grain to deform equally in a so-called upper-bound treatment where the stress is allowed to vary from grain-to-grain. With the additional constraint that some grains may deform differently than others, the self consistent model was developed to take grain interaction stresses into account as illustrated schematically in Figure S6.

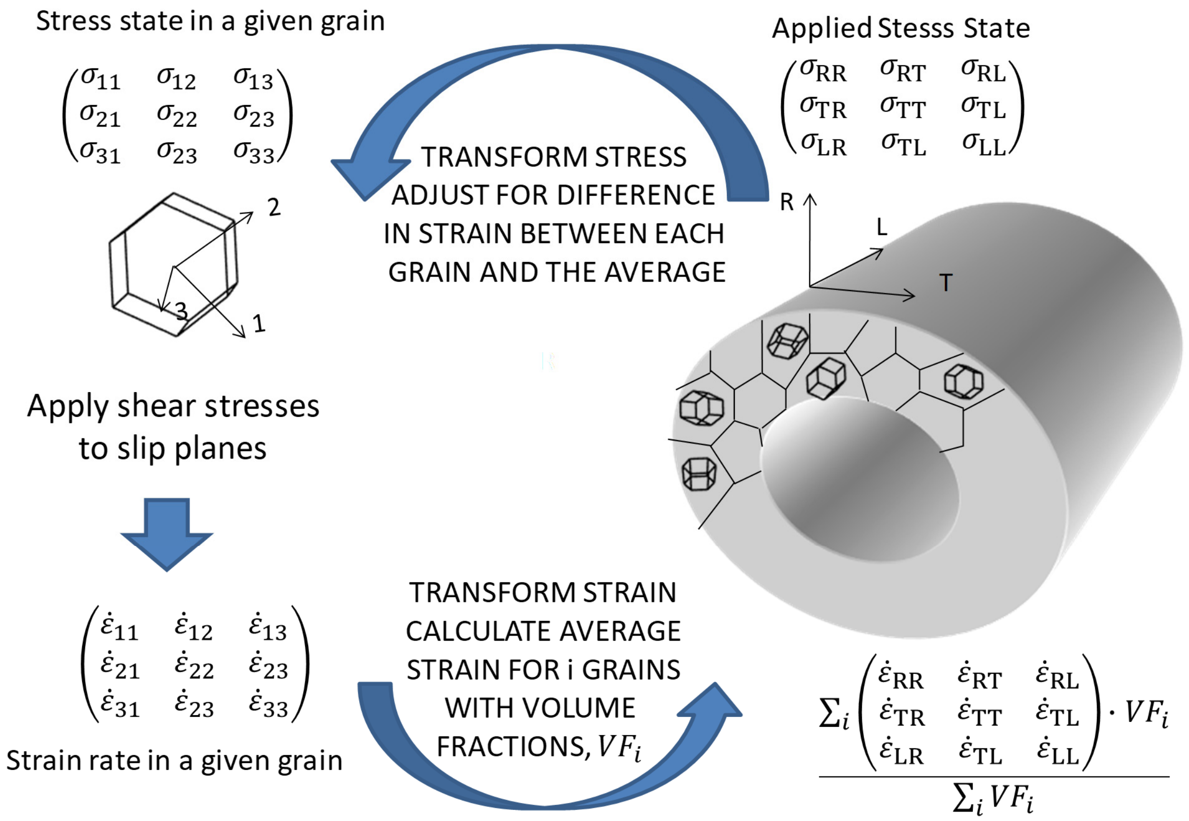

Figure S6. Determination of creep strain in a polycrystalline aggregate. The single crystal coordinates are represented by numerical suffixes and the component (bulk) coordinates are represented by alphabetical suffixes.

The first step in constructing polycrystalline model of this type is to describe the single crystal response during irradiation. The polycrystalline behaviour can then be calculated by resolving the combined strains from each grain according to their volume fraction and summing the result considering the grain interactions, as shown in Figure S6. Considering slip in a single grain Hutchinson [12] showed that one must first calculate the shear stress on the active slip plane, determine a strain rate for the simple shear and then transform this strain onto the crystal coordinate system. Assuming that the stress exponent for irradiation creep is one, the strain rate can be determined through a creep compliance tensor. Representing tensors by bold text, the single crystal deformation can be represented by [12],

$$\dot{\boldsymbol{\varepsilon}}^{\mathbf{c}}_{\mathbf{ij}} = \mathbf{k}^{\mathbf{c}}_{\mathbf{ijkl}}\boldsymbol{\sigma}^{\mathbf{c}}_{\mathbf{kl}} \quad (i, j, k \text{ and } l = 1, 2, 3) \tag{1}$$

where $\dot{\boldsymbol{\varepsilon}}^{\mathbf{c}}_{\mathbf{ij}}$ is the 2nd rank strain rate tensor in a single crystal;

$\mathbf{k}^{\mathbf{c}}_{\mathbf{ijkl}}$ is the 4th rank single crystal creep compliance;

$\boldsymbol{\sigma}^{\mathbf{c}}_{\mathbf{kl}}$ is the 2nd rank stress tensor in a single crystal.

At the single crystal level the components of strain can be assigned to different slip systems using the standard Schmid tensor formula. For a stress exponent =1, [11, 12], the creep compliance is,

$$\boldsymbol{k}_{ijkl}^{c} = \sum_{s} \dot{\gamma}_{0}^{s} \frac{\boldsymbol{m}_{ij}^{s} \boldsymbol{m}_{kl}^{s}}{\tau_{s}} \quad (2)$$

where $\dot{\gamma}_0^s$ is a reference (measured or calculated) shear strain of a slip system, *s*; τ_s is the critical resolved shear stress (measured or calculated) of the slip system, *s*; and $\boldsymbol{m}^s$ is the 2nd rank Schmid tensor characterizing the slip system in single crystal coordinates. Substituting equation (2) into equation (1), the term $m_{kl}^s \sigma_{kl}^c$ is the inner product of the Schmid and stress tensors referred to the same coordinate system, i.e. it is the sum of the product of the terms in m_{kl}^s with the terms in σ_{kl}^c having the same indices [12]). The inner product is a shorthand formula taking into account the tensor transformations needed to extract the shear stress on the slip system for a given stress state σ_{kl}^c in the crystal coordinate system. The shear stress on the slip system of interest then represents a scaling factor which, combined with the reference strain rate ($\dot{\gamma}_0^s$) and shear stress (τ_s) shown in Equation (2), and applied to the Schmid tensor (m_{ij}^s), which transforms the shear strain rate on the slip system back into the crystal coordinates, gives the strain rate tensor in crystal coordinates from slip on the slip system, s. The fourth rank compliance tensor ($\mathbf{k}_{\mathbf{ijkl}}^{\mathbf{c}}$) is then defined by the sum of the outer products ($\boldsymbol{m}_{ij}^s \boldsymbol{m}_{kl}^s$) as described in [12].

The relationships between the coordinate systems applicable to individual slip planes and the single crystal coordinate systems for the three crystal structures that apply to most reactor core materials are illustrated in Figure S7. Also shown is the Schmid tensor for slip on {111} in an FCC crystal using the notation described by Kelly and Knowles [13] for a symmetric shear strain tensor.

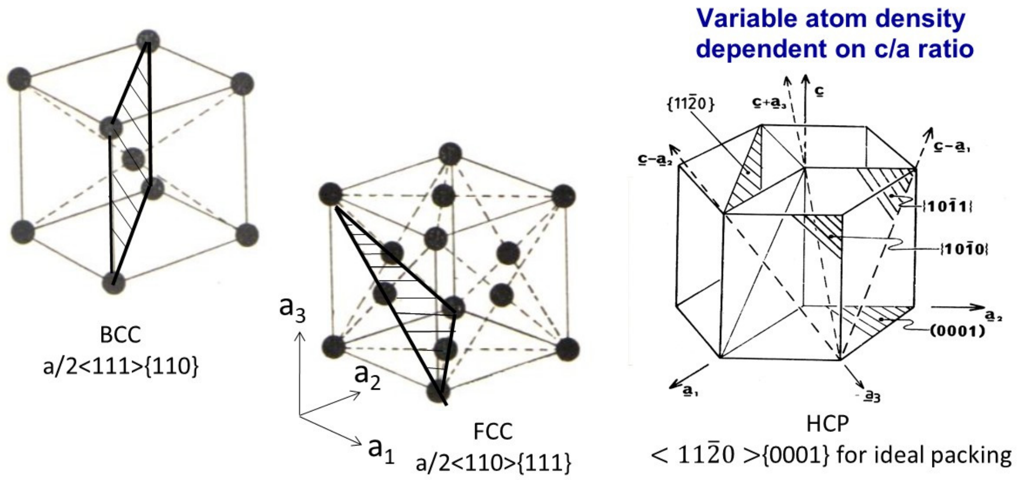

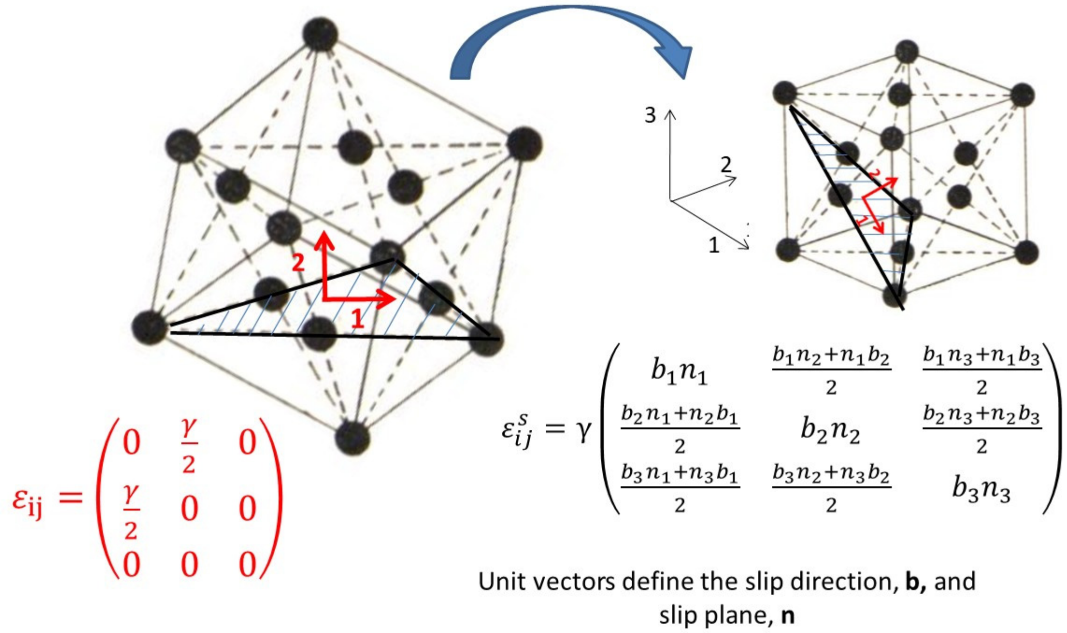

Figure S7. Relationship between the shear strain tensor for a given slip plane and the corresponding strain tensor (the Schmid tensor) in the single crystal coordinate system.

Kelly and Knowles showed how simple shears can be represented as a symmetric shear plus a rotation. The benefit of representing the shears as a symmetric tensor Figure S7) is that subsequent transformations give a symmetric tensor also, recognising that the rotations must be discarded and therefore represent an unphysical situation unless, of course, there was some real grain boundary rotation. By manipulating the tensors in such a way Kelly and Knowles could introduce a form for the Schmid tensor that can be applied in a general way to all slip systems using the Burgers' vector (b) and slip plane normal (n) while maintaining a symmetric form of the strain tensor in each case. The notation used by Kelly and Knowles shown in Figure S7 gives the same (Schmid) tensor as one would get from a formal tensor transformation of the symmetric shear tensor obtained by adding a rotation to the simple shear. Of course, if one is rigorous and applies the transformation to the simple shear the strain tensor in the single crystal coordinate system will be asymmetric. An example of the derivation of the Schmid tensor for one of the 12 slip systems in Zr (c+a slip on a pyramidal plane) is shown in Figure S8.

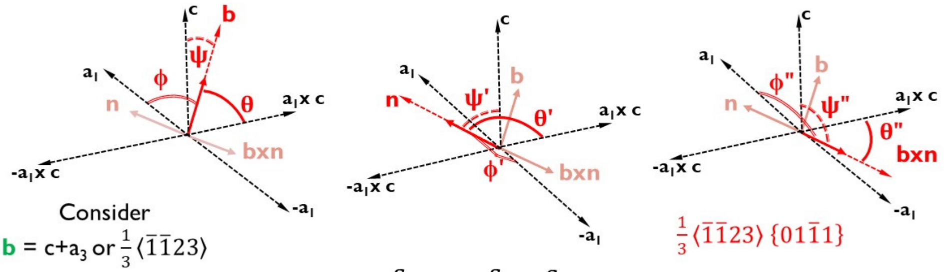

$$m_{ij}^{s} = a_{ij}^{s}\, a_{kl}^{s}\, \varepsilon_{kl}$$

$$a_{ij}^{s} = \begin{array}{c} \\ a_I \\ a_I\mathrm{x}c \\ c \end{array}\begin{array}{c} \begin{array}{ccc} b & n & b\mathrm{x}n \end{array} \\ \begin{bmatrix} \cos\phi & \cos\phi' & \cos\phi'' \\ \cos\theta & \cos\theta' & \cos\theta'' \\ \cos\psi & \cos\psi' & \cos\psi'' \end{bmatrix} \end{array} \qquad \varepsilon_{kl} = \begin{bmatrix} 0 & \gamma & 0 \\ 0 & 0 & 0 \\ 0 & 0 & 0 \end{bmatrix}$$

$$m_{ij}^{s}: \quad \varepsilon'_{ij} = \gamma \begin{bmatrix} \cos\phi'\cos\phi & \cos\theta'\cos\phi & \cos\psi'\cos\phi \\ \cos\phi'\cos\theta & \cos\theta'\cos\theta & \cos\psi'\cos\theta \\ \cos\phi'\cos\psi & \cos\theta'\cos\psi & \cos\psi'\cos\psi \end{bmatrix}$$

$$\begin{bmatrix} \phi & \phi' & \phi'' \\ \theta & \theta' & \theta'' \\ \psi & \psi' & \psi'' \end{bmatrix} = \begin{bmatrix} 105.41 & 90 & 164.59 \\ 62.60 & 151.49 & 97.28 \\ 32.10 & 61.49 & 103.5 \end{bmatrix} \qquad \varepsilon'_{ij} = \gamma \begin{bmatrix} 0 & 0.23 & -0.13 \\ 0 & -0.40 & 0.22 \\ 0 & -0.74 & 0.40 \end{bmatrix}$$

Figure S8. Derivation of the Schmid tensor for c+a slip on one slip system in Zr.

Woo's treatment of irradiation creep, [6, 11, 14], does not involve a rigorous derivation of the Schmid tensors as described by Groves and Kelly [15], but is similar in that it is constructed in such a way that the total deformation is comprised of orthogonal basis tensors that can be combined to describe any single crystal strain. The basis tensors employed by Woo are surrogates for the five independent slip systems necessary to describe an arbitrary volume-conserving deformation as outlined in [13]. It is important to emphasise that Woo's analysis does not apply to real independent slip systems, rather it is a mathematical construct.

In Woo's treatment the single crystal creep compliance tensor is obtained from the sum of the compliances derived from six basis tensors (denoted by the superscript λ or α) that substitute for the Schmid tensors for individual active slip systems, each weighted by a factor determined from experimental data. The basis tensors are described as if they were the result of shear strains that can be expected from a simple cubic but, in practice, are simply a means of assigning a strain to a stress having the same basis tensor. The net effect is that each individual basis acts as a viscous fluid – i.e. the strain basis has the same tensor components as the stress basis giving a resultant strain that is, in itself, isotropic, i.e. the strain basis tensor is proportional to the stress basis tensor. Anisotropy is introduced by weighting the strain response to each applied stress basis tensor. The basis tensors described in [11, 16], $\mathbf{b}^{\lambda}$, are not the same as the Schmid tensors characterizing each slip system described in [13, 15]. Rather than using tensors that correspond with the true slip systems of the hexagonal close-packed crystal, Woo chose slip systems for a simple cubic crystal. The hexagonal crystal has the same axes as the simple cubic basis but that is where any similarity ends. In this way Woo was able to describe the deformation of the hexagonal crystal as though it deformed subject to five independent slip systems of a simple cubic. In reality hexagonal close-packed crystals do not deform according to a simple cubic so the approximation employed by Woo cannot be considered rigorous. One basis tensor in particular ($\mathbf{b}^{1}$) represents the slip on pyramidal planes but is symmetric about the c-axis and therefore presumably applies to equal slip on all 12 pyramidal slip systems. To maintain

integrity (because one cannot expect that all 12 slip systems are always equally excited) this strain basis (as with all other strain basis tensors) can only be excited by the same stress basis. But $\mathbf{b^1}$ is the only basis representing pyramidal slip [9-11, 17]. Woo states that $\mathbf{b^1}$ also represents irradiation growth [11].

Although formulated and interpreted from a consideration of the strains arising from slip, Woo et al. point out that these basis tensors can be used to describe deformation by any mechanism [6, 11, 16]. In fact, the same set of basis tensors were employed to model irradiation creep based on diffusional mass transport [16]. Although they represent the strains due to slip on each slip system transformed into the crystal coordinates, they can also be used as the orthonormal basis on which to partition the contributions to the total strain of the single crystal where the strain arises from the elasto-diffusion tensor [16, 17]. The basis tensors and their relationship to the deformation of a simple cubic are shown in Figure S9 together with schematics showing the main slip planes for two grain orientations in Zr-2.5Nb pressure tubing – the intended application for Woo's analysis being a model for Zr-2.5Nb pressure tube deformation [9, 10].

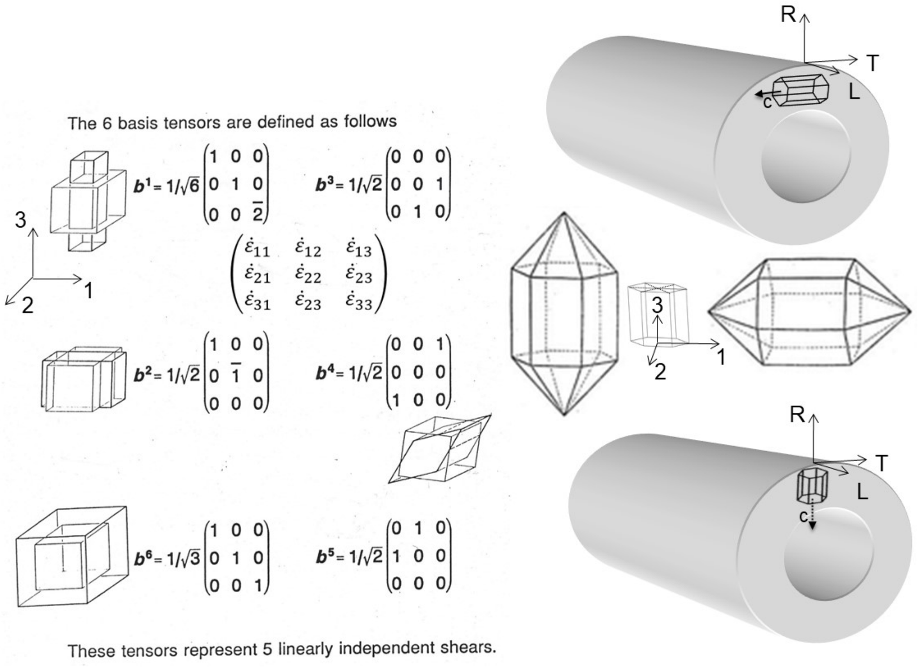

Figure S9. Basis tensors based on a simple cube used to construct a single crystal compliance tensor to represent deformation by slip on in Zr (shown by the hexagonal prism).

According to Woo et al., [11, 16], if one was to assign these basis tensors to slip, the Schmid tensors would represent slip on the planes shown in Figure S10.

Schmid Tensors
$b^{(1)}$ - represents <c+a> slip on $\{10\bar{1}1\}$
$b^{(2)}$ and $b^{(5)}$ - represent <a> slip on $\{10\bar{1}0\}$
$b^{(3)}$ and $b^{(4)}$ - represent <a> slip on (0001)

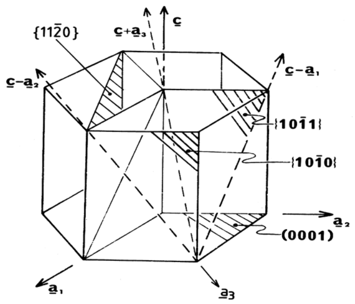

Figure S10. Basis tensors based on a simple cube used to construct a single crystal compliance tensor to represent deformation by slip on in Zr (shown by the hexagonal prism).

In Woo's analysis [11], the single crystal compliance ($\mathbf{K}^c$) is given by,

$$\mathbf{K}^{c}_{ijkl} = \Sigma_{\alpha} k_{\alpha}\, \mathbf{b}^{\alpha}_{ij}\, \mathbf{b}^{\alpha}_{kl} \tag{3}$$

and the single crystal strain is then given by,

$$\dot{\boldsymbol{\varepsilon}}^{c}_{ij} = \Sigma_{\alpha} \mathbf{k}_{\alpha}\, \mathbf{b}^{\alpha}_{ij}\, \mathbf{b}^{\alpha}_{kl}\, \boldsymbol{\sigma}_{\alpha} \mathbf{b}^{\alpha}_{kl} \tag{4}$$

where the stress tensor for each basis is,

$$\boldsymbol{\sigma}_{\alpha} = \boldsymbol{\sigma}_{\alpha} \mathbf{b}^{\alpha}_{kl} \tag{5}$$

where $\boldsymbol{\sigma}_{\alpha}$ and k_{α} are eigenvalues for each basis derived for the applicable stress and microstructure [9]. As Woo states "k_{α}, is the eigen creep compliance that causes the crystal to deform in the α th characteristic straining mode when subjected to a stress in the same characteristic mode". This is why the strains represented by the basis tensors, $\mathbf{b}^{\lambda}$, are only excited by stresses that have the same basis tensor. Using this formalism Woo was able to construct a single crystal compliance tensor, $\mathbf{K}^{c}_{ijkl}$, from a linear combination of basis tensors using a set of factors for k_{α}. In this way the single crystal compliance is simply defined by three eigenvalue parameters k_1, k_2, and k_3 that can be chosen to best represent the single crystal response of the material.

The applicability of a compliance tensor constructed in this way depends of how well the choice of k_{α} represents the true response of the material based on a rigorous Schmid tensor construction of the type described by Kelly and Knowles [13] and represented formally by Hutchinson [12]. The reference for the proof of this analysis given by Christodoulou et al. [9] is "Woo, C. H., Causey, A. R. and Holt,R. A., invited paper in "Diffusion and Defect Data - Solid State Data", 1990, in press" but does not appear to have been published. For this reason, the efficacy of what Woo has done remains to be established. Therefore, Woo's model can at best be considered an approximation and at worst can be considered not applicable to the deformation it is intended to simulate.

The reconciliation of a slip based model and a stress exponent of 1 is justified by the rate-determining, slip-enabling, step coming from radiation-enhanced climb of dislocations over barriers [2, 5]. In the model described in [10] the irradiation creep deformation is assumed to be by slip only and is therefore dependent on dislocation structure and texture only; it is assumed that the grain structure does not affect the deformation anisotropy. Such an approach would be an elegant and efficient way to describe the creep anisotropy, provided that the anisotropy is only dictated by the texture, i.e. dislocation slip, and provided one could conduct the right experiment to determine the single crystal creep compliance tensor. The best way to determine whether the polycrystalline model is valid, i.e. that the single crystal compliance is accurate and the anisotropy is only dictated by texture, is to compare with experiment. However, even that is difficult when the deformation model is comprised of different contributions other than irradiation creep for which one wants to derive the anisotropy factors (F, G and H) for the polycrystal [9, 10].

Whereas the creep compliance in any given direction for a given stress state can be deduced from the slope of the creep rate as a function of stress, for the model described by Holt [18] and Christodolou et al. [9, 10] both thermal creep and irradiation creep are active and one can only separate the two using another model. The best that one can do is either subtract an estimate of thermal creep or assume that it is negligible, as in the case of irradiation creep experiments at low temperatures and high neutron fluxes [19]. With this caveat, the data on irradiation creep rate can then be used to establish a single crystal creep compliance in the context of the polycrystalline model described by Christodoulou et al., [10]. A validation of the creep anisotropy factors described in Christodoulou et al., has been presented by Causey et al., [20] and is reproduced in Figure S11(a). It has been claimed that the model works well [18], however some of the key validation data cited [21], i.e. reference 17 in [20], and circled in Figure S11(a), cannot be verified from the information available in [21].

The polycrystalline model outlined in [10] shows reasonable agreement with the OSIRIS data for a narrow range of textures corresponding to the OSIRIS creep capsules (the two data points at f_T-$f_R \sim 0.2$), Figure S11(a), for which k_1, k_2 and k_3 were chosen [9]. Although there is a clear indication that the creep anisotropy is affected by texture, it has been assumed that the anisotropy is dependent on the texture only [9, 10, 18]. This is largely based on the underlying assumption that the irradiation creep is dominated by dislocation slip. We know from irradiation growth that grain structure changes the growth anisotropy [18], so it is hardly surprising that grain structure also affects the creep anisotropy [3]. For the pressure tube deformation equation, Christodoulou et al. [10] included the effect of grain size empirically through the inclusion of the $K_4(x)$ parameter, which is meant to capture variations in microstructure other than texture along the pressure tube length. $K_4(x)$ also accounts for any variation in texture not adequately accounted for by the polycrystalline model. The introduction of $K_4(x)$ renders the anisotropy factor $C_4(x)$ redundant, unless it can be shown that the empirical equation obtained for one orientation of the component (in this case the axial component) can be applied to other directions through the anisotropy factor $C_4(x)$, i.e. unless it can be shown that $K_4(x)$ is a magnitude effect and is the same for any orientation of the component. There is no reason to assume that this is true.

One problem with the data shown in Figure 11(a) is that one is mixing data for an irradiation (in NRU) where thermal creep is deemed to have a significant contribution to the strain with data for an irradiation (in OSIRIS) where the creep is deemed to be dominated by irradiation creep. Another problem is that the data were averaged for several creep capsules with slightly different textures and irradiated at different temperatures. The data for individual tubes irradiated in OSIRIS [19] has been plotted together with more recent data from NRU [22] in Figure 11(b). In the model of Christodoulou et al. [10] it was assumed that the anisotropy is independent of temperature, but later work from irradiation in NRU [22] has shown that this assumption is incorrect (also shown in Figure S11(b)).

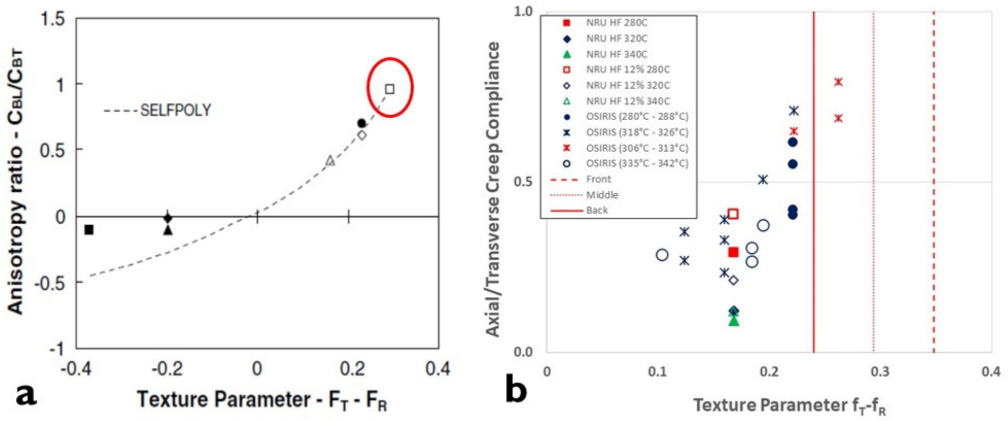

Figure S11. (a) Comparison of measured and calculated (from texture) anisotropy ratios (axial strain rate/transverse strain rate) for internally pressurized tubes under irradiation creep conditions. The open square represents CANDU pressure tubes. f_T and f_R are the Kearns' basal pole texture parameters [21]. Reprinted/adapted with permission from [18]. 2019, Elsevier; (b) Dependence of the ratio of biaxial in-reactor creep compliance in the transverse and axial directions of Zr-2.5Nb pressure tubing on the texture parameter, ft – fr [19, 22].

The main shortcomings of models that assume I-creep, where dislocation slip is the dominant deformation mechanism, are: (i) slip may not be the dominant mechanism; (ii) there is no provision for diffusional mass transport to contribute to the strain other than through enhancing climb of dislocations over obstacles. If the irradiation creep was dominated by slip one would eventually expect to see two characteristics in the observed deformation behaviour: (i) the creep would eventually cease as the mobile dislocations are exhausted at grain boundaries or at large obstacles (large inclusions, precipitates or voids for example), and (ii) the total creep and the creep rate would be smaller in small-grained material as the dislocation travel shorter distances before running into boundaries. In fact, neither of these characteristics are demonstrated by irradiated Zr-alloys at CANDU reactor operating temperatures. In the absence of evidence for irradiation creep cessation in Zr-alloys, and given that diametral creep increases with decreasing grain size [3, 9, 10, 23], the evidence suggests that a mass transport mechanism is more applicable. There are other reasons why there are shortcomings with slip-based models and this

stems from the need to separate the strain arising directly from diffusional mass transport and the strain arising from slip.

Although many of the cold-worked dislocations are locked in sessile junctions and do not appear to change during irradiation [2], some dislocations will be mobile and these could then potentially contribute to creep strain from slip. However, post-irradiation annealing of Zr-2.5Nb pressure tubing irradiated to fluences of 8 x 10^{25} n.m^{-2} removes the radiation damage to reveal a microstructure that is close to the original cold-worked structure [24] indicating that little, if any, of the network dislocations have moved, otherwise there would be a visible reduction in dislocation density as the dislocations glide and annihilate at grain boundaries. Based on the available evidence one can conclude that irradiation creep in Zr-2.5Nb pressure tubing is not dominated by dislocation slip, i.e. irradiation creep is not governed primarily by a climb-and-glide type mechanism. However, in the theoretical analysis of polycrystalline creep by Woo and So [16] the creep due to elasto-diffusion was determined to be an order of magnitude lower than creep by slip. They concluded that either the slip-based polycrystalline model [9] was dominant, or the model parameters for diffusion were incorrect. Grain boundaries were not included in their analysis, and it is hardly surprising that the amount of strain one can obtain from dislocation slip turned out to be more than the strain possible from dislocation climb, as they concluded.

When grain boundaries are incorporated in a rate-theory model including a bias due to stress, it is relatively easy to show that one can account for irradiation creep by selecting appropriate bias parameters (equivalent to anisotropy factors). Even the parameters in the slip-based models at the single crystal level are adjusted so that the polycrystalline output fits the experimental data [9]. For creep capsules and pressure tubing all of the creep giving a c-axis strain is attributed to pyramidal slip [9]. However, there is no evidence for dislocation mobility or that there are even sufficient mobile c-component dislocations to generate the necessary strain to match the data. It should be re-emphasised that prismatic dislocation loops can not provide a source for mobile dislocations that can contribute to creep strain by slip even if they have evolved by climb into a coarse network [5, 25, 26]; the strain resolved in the direction of the Burgers' vector of a dislocation loop is the same whether or not it has moved along its glide cylinder or even if it tilts.

S3 Modelling Irradiation Creep using Rate Theory

During irradiation equal numbers of interstitial and vacancy point defects are produced by atomic displacements by neutrons. These irradiation-induced point defects have steady-state concentrations that are many orders of magnitude higher than the thermally-induced point defects at temperatures up to about 500 °C. The point defect concentrations can be expressed as numbers of defects per unit volume, but it is often easier (and more elegant) to express the concentrations as atom fractions. Therefore, instead of N point defects per unit volume one expresses the fractional concentration as $N \cdot \Omega$, where Ω is the atomic volume and $\frac{1}{\Omega}$ is the number of atoms per unit volume. The number of atomic displacements is expressed similarly as displacements per atom (dpa). Considering the strain arising from the annihilation of these point defects at a given sink, one can envisage an interstitial as adding a positive strain in the direction of either the Burgers vector for a dislocation, or the surface normal for a surface such as a cavity or grain boundary. Likewise, a vacancy annihilating at a given sink produces an opposite strain

to the interstitial at the sink. For a cavity/void the strain is only manifested macroscopically by the atoms that are not in the cavity and is thus always positive.

The strain rate at a surface or a dislocation is equal to the flux of point defects expressed as an atom fraction. Consider a unit cube with N atoms along each edge. The total number of atoms is N^3. If 1% of the atoms (expressed as an atom fraction) are displaced and migrate to one of the surfaces, then the number of displaced atoms arriving at that surface is $0.01 \cdot N^3$. The number of atom sites on the surface is N^2 and the fractional coverage is then $0.01 \cdot N^3 / N^2 = 0.01 \cdot N$. But the cube is N atoms in length perpendicular to each face. Therefore, the strain is $0.01 \cdot N/N = 0.1$, i.e. the strain from a net flux of point defects expressed as an atom fraction of 0.01 or 1% is therefore 1%. Creep strains from diffusional mass transport are thus calculated by computing the net flux (balance of interstitials and vacancies) to each sink responsible for the strain in each direction.

S3.1 Rate Equations

For freely migrating point defects, the diffusion to different sinks can be described by a set of simultaneous rate equations,

$$\phi - \sum_s k_v^2 \, D_v C_v - aC_i C_v = 0 \tag{6}$$

$$\phi - \sum_s k_i^2 \, D_i C_i - aC_i C_v = 0 \tag{7}$$

where (ϕ) is the freely-migrating point defect generation rate, ($k_{i,v}^2$) is the sink strength summed over all sinks (s) and has units of m^{-2}, ($D_{i,v}$) is the diffusion coefficient and has units of $m^2.s^{-1}$, ($C_{i,v}$) is the fractional point defect concentration in the diffusive medium and (a) is a recombination rate parameter that is proportional to the interstitial diffusion coefficient, D_i [27, 28]. The point defects are designated (i) for interstitial atoms, and (v) for vacancies. The rate of flow of point defects to a given sink is determined by the concentration difference between the steady-state concentration in the medium (mid-way between sinks), (C_α) and the concentration at the sink surface (C_α^{th}). The thermal emission term (C_α^{th}) is relatively insignificant for most structural materials at power reactor operating temperatures and only becomes important at high temperatures, and then only for vacancies, when the equilibrium vacancy concentration becomes comparable with the steady-state concentration due to neutron displacement damage.

Following Heald and Speight [28] the effect of recombination is given from the solution of the two simultaneous equations (1) and (2),

$$k_\propto^2 \, D_\propto C_\propto = \phi \, F(\eta) \tag{8}$$

$$F(\eta) = \frac{2}{\eta}\{(1+\eta)^{0.5} - 1\} \tag{9}$$

$$\eta = \frac{4\, a\, \phi}{D_v \, D_i \, k_v^2 \, k_i^2} \tag{10}$$

As radiation damage produces equal quantities of interstitial (i) and vacancy (v) point defects (denoted by the subscript α), the net atom fraction flux of each type of point defect to all sinks is equal to the generation rate in dpa, thus giving a mass balance for the displacement damage.

This mass balance only applies to vacancies and interstitials created by direct atomic displacement. Other point defects such as He or H created by transmutation reactions are added to the system and the only mass balance to consider is the one balancing production with annihilation at sinks. In such cases annihilation often changes the nature of the sink (e.g. increasing the pressure inside a cavity). The total number of vacancy and interstitial point defects available to diffuse to the point defect sinks after mutual recombination is given by $\phi\, F(\eta)$. The mutual recombination, represented by $F(\eta)$, is a function of the sink strengths (k_α), the freely migrating point defect production rate (ϕ), which is the atom fraction of displaced atoms that survive spontaneous recombination or clustering within a collision cascade [27], the diffusion coefficients (D_α) and mutual recombination parameter (α). Those point defects of opposite sign that migrate to the same sink result in no net mass transfer. The net accumulation of one sign or the other causes a change in the size of the sink (for clusters) and can alter the properties of the sink, e.g. pinning of screw dislocations by helical climb or jog formation.

The net flux of point defects to a sink of a given type (s), or orientation, can then be calculated as,

$$J_s = (k_i^2)_s\, D_i\left(C_i - C_i^{th}\right) - (k_v^2)_s\; D_v\left(C_v - C_v^{th}\right) \qquad (11)$$

where the point defect thermal emission term is now added. Thermal emission is only important at high temperatures. For a given displacement damage rate as the temperature rises the thermal emission terms will be increasingly dominant. Eventually the effect of irradiation will be insignificant compared to the effect of temperature in producing point defects.

The sink strength (k) is a measure of the probability that a point defect will annihilate at a given sink. This will be determined by the density and orientation of said sinks and, in the case of dislocations, the elastic interaction between the strain field around the sink and the point defects [28]. As we are dealing with balance equations it is only necessary to be able to compute the relative sink strengths for the different sinks. To do so, it is helpful to consider what the sink strength represents physically. If L is the average distance a point defect moves before it encounters a sink, according to random-walk theory [27] the average distance (L) that a point defect migrates in n randomly oriented jumps with a jump distance (r) is given by,

$$L^2 = n\,.\,r^2 \qquad (12)$$

The relationship between the atomic jump frequency (ω) of a given defect, the jump distance (r), and the diffusion coefficient (D) is,

$$D = \alpha\,.\,\omega\,.\,r^2 \qquad (13)$$

The α is a geometric factor (assumed = 1 for a vacancy diffusing in an fcc lattice [27]). The rate at which a vacancy point defect will move a distance L is then simply the ratio of the number of jumps per second (ω) and the average number of jumps (n) to move a distance L, i.e. $\frac{D}{L^2}$.

The diffusion length (L) is inversely proportional to the sink density (ρ) and the rate of annihilation of point defects at a given sink, for which the diffusion length L applies, is

proportional to $\frac{D}{L^2}$ or Dρ, where ρ is the sink density, which for dislocations is in units of m/m^3. The rate at which point defects annihilate at sinks is then given by the ratio of the mean squared distance travelled per second (proportional to D) and the square of the mean distance travelled to reach a sink (L^2) multiplied by the fractional concentration of the point defect in question (C),

$$Rate \propto \frac{D \cdot C}{L^2} \qquad (14)$$

The value of L is inversely proportional to the sink density but is not simply the distance between sinks. There are other factors, for example strain field interactions, that affect the probability that the point defect reacts with a given sink. For dislocations the rate can be expressed as,

$$Rate = z \cdot \rho_d \cdot D \cdot C \qquad (15)$$

Olander [27] considers z to be a parameter equal to the number of trapping sites surrounding the dislocation where the point defect is spontaneously captured and is similar to the trapping cylinder calculated by Heald and Speight [28]. For most practical purposes there are only three types of sinks to consider: (i) dislocations, (ii) cavities, and (iii) grain boundaries. The derivation of the sink strengths assuming diffusion-limited kinetics is described for each.

S3.1.1 Sink Strength for Dislocations

By solving diffusion equations with cylindrical geometry, Heald and Speight [28] showed that the rate constant for point defect interactions with dislocations is

$$k_{i,v}^2 = \frac{1}{L_d^2} = z_{i,v}\rho_d \qquad (16)$$

where ρ_d is the dislocation density of a given type of dislocation, and

$$z_{i,v} = \frac{2\pi}{\ln\frac{2R}{l_{i,v}}} \qquad (17)$$

where *R* is the mean distance between dislocations and $l_{i,v}$ is determined from the elastic strain field interactions between the dislocation and respective point defects. One can think of $l_{i,v}$ as the radius of an effective trapping cylinder [28].

S3.1.2 Sink Strength for Cavities

By solving diffusion equations with spherical geometry, Heald and Speight [28] also calculated the sink strengths for cavities. Cavities are considered "neutral" sinks because, unless highly pressurized, the strain field is not large compared to the cores of dislocations. Accordingly, the same reaction rate expression is applicable for both interstitials and vacancies.

$$k_{i,v}^2 = \frac{1}{L_c^2} = 4\pi r_c \rho_c \qquad (18)$$

where r_c is the cavity radius and ρ_c is the cavity number density (number per cubic metre).

When cavities are pressurized, it is assumed that the main influence of the pressurization is in the absorption and emission of vacancies from the cavity surface. Apart from the free energy of the vacancy itself, the free energy change of the cavity caused by the absorption of one vacancy is given by -($p - \frac{2\cdot\gamma}{r}$)Ω, where p is the pressure in the cavity, γ is the surface energy of the cavity, r is the cavity radius and Ω is the atomic volume [29].

S3.1.3 Sink Strength for Grain Boundaries

Grain boundaries are a special case. Although the high densities of cavities and dislocations within the matrix can be assigned average sink strengths and the partitioning of point defects between those sinks can be calculated assuming no other interactions, the flux of point defects to the grain boundaries surrounding the sinks within the grain is determined by the mean steady-state concentration in the interior and the concentration gradient at the boundary. As the steady-state point defect concentration in the matrix is a function of the total sink density within the grain, the grain boundary sink strength is not only dependent on the grain boundary density (grain size) but also on the density of other sinks within the grain interior.

The sink strengths of grain boundaries have been calculated assuming spherical geometry (treating the grain as a sphere of diameter *d*), while considering the internal sinks within the grains [30, 31]. The diffusion of point defects to the grain boundary is determined from the steady-state point defect concentration within the grain interior and the concentration gradient at the boundary, assuming that the boundary itself is a perfect sink. As with the cavities, the same reaction rate expression is applicable for both interstitials and vacancies. If the average diffusion length averaged over all sinks is λ (and the average sink strength, $k = \frac{1}{\lambda}$) then the sink strength for the grain boundaries are given by [31],

$$k_{i,v}^2 = \frac{6}{\lambda d}, \text{ or } \frac{6k}{d}, \text{ when } \ (z_{i,v}\rho_d + 4\pi r_c\rho_c)^{0.5} > 1/d \tag{14}$$

when the diffusion length for the interior is very much smaller than the grain boundary dimensions. Alternatively,

$$k_{i,v}^2 = \frac{24}{\lambda d}, \text{ or } \frac{24k}{d}, \text{ when } \ (z_{i,v}\rho_d + 4\pi r_c\rho_c)^{0.5} < 1/d \tag{15}$$

when the diffusion length for the interior is very much larger than the grain boundary dimensions.

S3.2 Balance Equations

The physics behind the sink strengths that dictate the rate at which a given point defect will migrate to and be absorbed by a given sink can be simplified if one considers that one is aiming to determine the relative probabilities of absorption for each sink. One can then create a set of balance equations for the flow of point defects created by irradiation to all of the sinks based on relative probabilities. In this respect the rate of point defect production is important rather than the speed at which the defects migrate to the sinks because the production rate is typically slower than the rate at which the point defects get to a sink. At sufficiently high temperatures in power reactors, 250 °C - 350 °C for example, one can consider that a point defect migrates to a sink

almost instantaneously compared to the time it takes for another point defect to be created and migrate to the same sink. This is the so-called sink-dominated regime. At high temperatures (> 500 °C for example) the thermal equilibrium vacancy concentration is comparable to the steady-state concentration of point defects produced by irradiation and thermal processes dominate, except when He is generated [32]. At low temperatures the steady state concentration of the slower-moving vacancies (those with a high migration energy) increases together with the rate of mutual recombination with interstitial point defects. The temperature regime where recombination is important is called the recombination-dominated regime and is typically < 200 °C for austenitic alloys and < 100 °C for Zr-alloys, the latter having a lower vacancy migration energy [2]. In the sink-dominated regime any temperature dependence in a rate theory calculation is the result of variations in the sink densities with temperature. For all practical purposes mutual recombination can be ignored in the sink-dominated regime. One can also formulate balance equations by applying suitable bias factors for different sinks that dictate the relative probability of one type of defect being absorbed compared to another.

For a given range of stresses, temperatures and neutron fluxes/fluences, one can assume that irradiation creep to a large extent is governed by the same processes that control irradiation growth, i.e. diffusional mass transport. It is reasonable then to consider that irradiation growth is an intrinsic response of the material to neutron irradiation that is represented by a suitable bias factor for each sink. The bias factor may reflect the tendency for interstitials to be absorbed at dislocations [30] or the probability that a boundary absorbs different point defects according to its orientation in an anisotropic diffusive medium [17]. These intrinsic bias factors are modified by the imposition of a stress. In the context of a simple mass transport model, involving dislocations only, the effect of stress can be included as a bias factor that is in addition to the interstitial bias factor. As an example, Figure S12 is an idealised schematic showing a simple model for large-grained cold-worked Zircaloy-2 fuel cladding subject to a uniaxial stress during irradiation. For simplicity the strain from a-type dislocations and dislocation loops has been resolved into two orthogonal directions in the basal plane. The creep rate in the direction of the uniaxial tensile stress (a_1) is determined by calculating the interstitial and vacancy flux to sinks aligned to give strain in the direction a_1, as illustrated. This simple example illustrates that rate theory balance equations are simply constructed so that the point defect generation rate is balanced by the point defect fluxes to the different sinks. If the sum of the interstitial fluxes to all sinks is given by G, the sum of the vacancy fluxes to all sinks is -G and thus the total flux to all sinks is zero. One can extract the flux (expressed as a strain rate) to a given sink type. In this example the flux to sinks giving strain in the direction a_1 is shown in the 3-D plot. The strain is a function of all the sink strengths and the locus of the maximum strain is given by the peak in the 3-D plot.

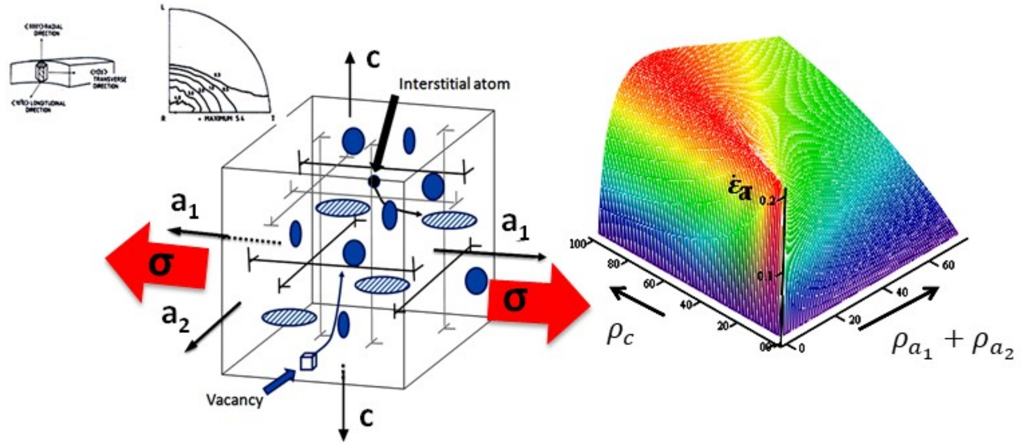

$$Ja_1 = \dot{\varepsilon}_{a_1} = \left[\frac{(1+b+s).\rho a_1}{(1+b+s)\rho a_1 + (1+b).\rho a_2 + \rho_c} - \frac{\rho a_1}{\rho a_1 + \rho a_2 + \rho_c}\right] \cdot G$$

$$Ja_2 = \dot{\varepsilon}_{a_2} = \left[\frac{(1+b).\rho a_2}{(1+b+s).\rho a_1 + (1+b).\rho a_2 + \rho_c} - \frac{\rho a_2}{\rho a_1 + \rho a_2 + \rho_c}\right] \cdot G$$

$$Jc = \dot{\varepsilon}_c = \left[\frac{\rho_c}{(1+b+s).\rho a_1 + (1+b).\rho a_2 + \rho_c} - \frac{\rho_c}{\rho a_1 + \rho a_2 + \rho_c}\right] \cdot G$$

Figure S12. Schematic and rate-theory output showing irradiation growth model results for the longitudinal direction of a large grained Zircaloy-2 as a function of the a (ρ_a) and c (ρ_c) dislocation densities (sink strengths). The interstitial bias factor for a-type dislocations is b. The stress bias factor is given by s. The c-component dislocations (loops) are assumed to be neutral.

As stated previously the strain may arise directly from the point defects themselves when they annihilate at an oriented sink or may involve the activation of climb and glide. Without a direct measure of the effect of stress on point defect diffusion one has to decide which mechanism dominates from empirical data. It has been shown that for pressure tubing at low stresses and temperatures mass transport is the most likely mechanism simply because creep from SICG cannot be reconciled with all the available evidence. Dislocation slip is important at low fluences, either during primary creep or at low neutron fluxes when the contribution from mass transport is small. Dislocation slip also appears to be important when the radiation damage density is low and there is increased thermal activation of slip at high irradiation temperatures [22]. Of course, dislocation slip will also dominate at stresses high enough that yielding occurs, albeit at a higher stress than for unirradiated material. When considering irradiation creep at moderate reactor operating temperatures (250 °C – 350 °C) and stresses (< 150 MPa), one must consider that the mechanism is dominated by diffusional mass transport directly and not just by enabling glide. In this context one can envisage the irradiation creep of Zr-2.5Nb pressure tubing to be an extrinsic effect, i.e. a modification of the intrinsic irradiation growth by the applied stress.

Even when the creep can be explained by dislocation slip, mass transport is still a controlling factor and may be considered rate-determining given that pre-irradiated material exhibits low

out-reactor creep rates compared with unirradiated material and compared to irradiated material in reactor, provided the neutron flux is high enough [22]. The creep rate of irradiated material only exceeds that of an out-reactor test once the neutron flux (displacement damage rate) is sufficiently high that the creep is dependent on either climb and glide or simple mass transport. The key to understanding irradiation creep is to know the state of the material microstructure (as fabricated and during irradiation). One can then decide which creep mechanism likely applies and assess the behaviour accordingly. For Zr-2.5Nb pressure tubing at nominal CANDU reactor operating conditions one can conclude that diffusional mass transport involving annihilation of point defects at grain boundaries is the most likely mechanism for irradiation creep. Using rate theory one can explore the effect of changes in an idealised grain structure to tailor the response to give the dimensional changes that are desired, for example lower elongation or lower diametral strain [3]. Figure S13 shows calculated strain rates for radial, longitudinal and transverse directions in a pressure tube as a function of axial location for nominal fluxes, stresses and temperatures applicable to pressure tubes in a CANDU reactor [3]. The tube is assumed to have a fixed transverse basal texture as depicted with a stress bias (s) comparable with the dislocation bias for dislocations (b). Results for two grain shapes are shown. The model shows that a tube that has grains with short dimensions in the transverse direction will exhibit low axial but high diametral creep. For Zr-2.5Nb pressure tubes the grains have a large variety of shapes and orientations and therefore models need to be developed that account for these variations [2].

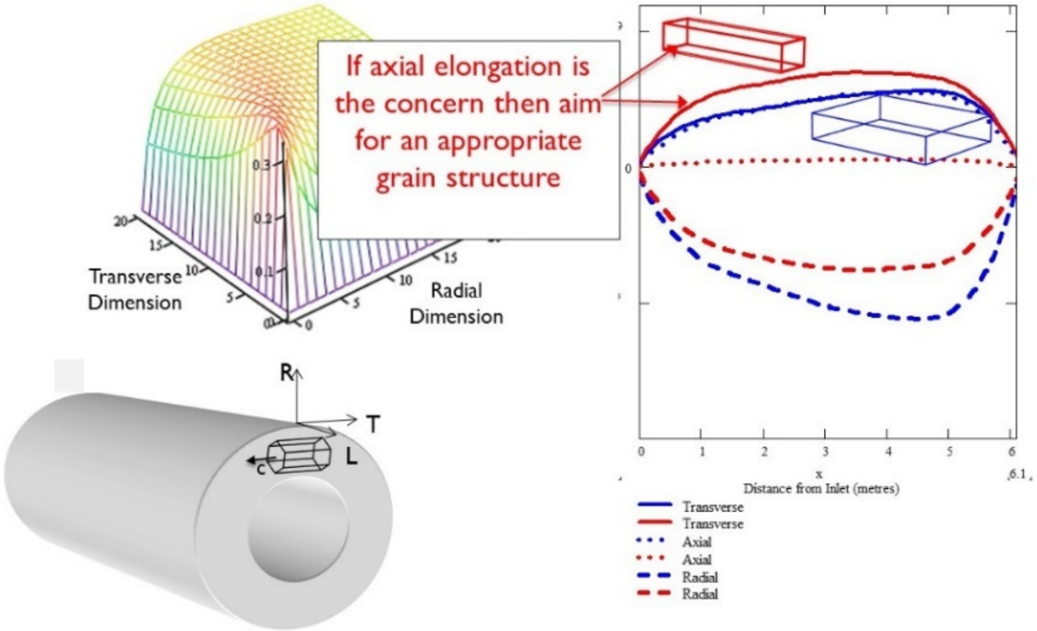

Figure S13. Schematic illustrating the relationship between grain dimensions and calculated strains for a fixed grain orientation and dislocation structure using rate-theory applied to Zr-2.5Nb pressure tubing. The grain boundaries compete with network dislocations and dislocation loops as sinks for point defects and the resultant strain anisotropy is therefore dictated by the size and shape of the grains [3].

Supplementary Material - References

1. Kearns, J.J., Thermal Expansion and Preferred Orientation in Zircaloy, WAPD-TM-472, 1965.
2. Adamson, R.B.; Coleman, C.E.; Griffiths, M., Irradiation creep and growth of zirconium alloys: A critical review, J. Nucl. Mater. 2019, 521, 167-244.
3. Griffiths M., Bickel G.A., DeAbreu R., Li, W., Irradiation Creep of Zr-Alloys, in Mechanical and Creep Behavior of Advanced Materials, The Minerals, Metals & Materials Society, I. Charit et al. (eds.), The Minerals, Metals & Materials Series, 2017.
4. Fleck, R. G., Price, E. G., and Cheadle, B. A., Pressure Tube Development for CANDU Reactors, Zirconium in the Nuclear Industry: Sixth International Symposium. ASTMSTP824, D. G. Franklin and R. B. Adamson, Eds., American Society for Testing and Materials, pp. 88-105 1984.
5. Onimus, F.; Jourdan, T.; Xu, C.; Campbell, A.A.; Griffiths, M. Irradiation Creep in Materials; Comprehensive Nuclear Materials; Konings, R., Ed.; Elsevier: Amsterdam, The Netherlands, 2020; Volume 1–10, pp. 310–366.
6. Woo C. H., Causey A. R. and Holt R. A., Methods of analysis and measurement of irradiation creep in non-cubic metals, Phil. Mag. A, Vol. 79, No. 1, pp.59-84, 1999.
7. Coleman, C. E., Causey, A. R., and Fidleris, V., In-reactor creep of zirconium--2. 5 wt% niobium at 570 K, J. Nucl. Mater. 60 (1976) 185-194.
8. Holt, R. A., Causey, A. R., and Fidleris, V., Dimensional stability and mechanical behaviour of irradiated metals and alloys, Proceedings of the International Conference on Dimensional Stability and Mechanical Behaviour of Irradiated Metals and Alloys, Vol. 1, (London: British Nuclear Energy Society), 1983, p. 175.
9. Christodoulou, N., Causey, A. R., Woo, C. H., Tome, C. N., Klassen, R. J., and Holt, R. A., Modelling the Effect of Texture and Dislocation Structure on Irradiation Creep of Zirconium alloys, Effects of Radiation on Materials: 16th International Symposium. ASTM STP 1175, A.S. Kumar, D.S. Gelles, R.K. Nanstad, and E.A. Little, Eds., ASTM Philadelphia, pp. 1111-1128, 1993.
10. Christodoulou N. Causey A. R., Holt R. A., Tomé C. N., Badie N., Klassen R. J., Sauvé R. and Woo C. H., Modelling in-reactor deformation of Zr-2.5Nb pressure tubes in CANDU power reactors, Zirconium in the Nuclear Industry: Eleventh International Symposium, ASTM 1295, E.R. Bradely and G. P. Sabol, Eds. American Society for Testing and Materials, Philadelphia, pp. 518-537, 1996.
11. Woo C. H., Polycrystalline Effects on Irradiation Creep and Growth in Textured Zirconium, J. Nucl. Mater. Vol. 131, pp. 105-117, 1985.
12. Hutchinson, J.W., "Bounds and Self-Consistent Estimates for Creep of Polycrystalline Materials", Proc. R. Soc. Lond. A. 348, pp. 101-127, 1976.
13. Kelly, A.F. and Knowles, Crystallography and Crystal Defects, Wiley, 2012.
14. Woo C.H., Effects of Anisotropic Diffusion on Irradiation Deformation, ASTM STP 955, pp. 70-89, 1987.
15. Groves, G.W. and Kelly, A., Independent slip systems in crystals, The Philosophical Magazine: A Journal of Theoretdaviesical Experimental and Applied Physics, 8:89, 877-887, DOI: 10.1080/14786436308213843, 1963.
16. Woo C. H. and So C. B., Effect of stress on point defect diffusion in HCP metals and irradiation creep, Phil. Mag. A, Vol. 80, No. 1, pp. 1299-1318, 2000.
17. Woo C. H., Theory of Irradiation Deformation in Non-Cubic Metals: Effects of Anisotropic Diffusion, Journal of Nuclear Materials 159, 237-256, North-Holland, Amsterdam, 1988.
18. Holt R.A., In-reactor deformation of cold-worked Zr-2.5Nb pressure tubes, J. Nucl. Mater. 372 (2008) 182-214.
19. Walters L., Bickel G. A. and Griffiths M., The Effects of Microstructure and Operating Conditions on Irradiation Creep of Zr2.5Nb Pressure Tubing, Zirconium in the Nuclear Industry: 17th International Symposium, ASTM STP 1543, R.J. Comstock, P. Barberis, Eds, ASTM International, West Conshohocken, PA, pp. 693-725, 2015.
20. Causey A. R., Elder J. E., Holt R. A. and Fleck R. G., On the Anisotropy of in-reactor creep of Zr-2.5Nb tubes, in: Proceedings of the 10th International Symposium on Zirconium in the Nuclear Industry, ASTM STP 1245, pp. 202-220, 1994.
21. Causey A. R., Fidleris V., MacEwen S. R., Schulte C. W., In-reactor Deformation of Zr-2.5Nb Pressure Tubes, ASTM Spec. Techn. Publ. 956, pp. 54-68, 1988. DOI: 10.1520/STP25640S.Holt R.A., In-reactor deformation of cold-worked Zr-2.5Nb pressure tubes, J. Nucl. Mater. 372 (2008) 182-214.
22. DeAbreu, R.F., Bickel, G.A., Buyers, A.W., Donohue, S.A., Dunn, K., Griffiths, M., Walters, L., Temperature and neutron flux dependence of in-reactor creep for cold-worked Zr-2.5Nb, Zirconium in the Nuclear Industry: Eighteenth International Symposium, ASTM STP 1507, pp. 938-964, 2018.
23. Griffiths M., Davies W. G., Causey A. R., Moan G. D., Holt R. A. and Aldridge S. A., Variability of In-Reactor Diametral Deformation for Zr-2.5Nb Pressure Tubing, Zirconium in the Nuclear Industry: Thirteenth

International Symposium, ASTM STP 1423, G. D. Moan and P. Rudling, Eds., ASTM International, pp. 796-810, West Conshohocken, PA, 2002

24. Coleman C.E., Chow C.K., Ells C.E., Griffiths M., Ibrahim E.F., Sagat S., Rejuvenation of Fracture Properties of Irradiated Zr-2.5Nb by Heat Treatment, Proc. 15th Int. Symp. on the Effects of Irradiation on Materials, ASTM STP 1125 (1992) 318-336.
25. Garner F.A., Irradiation performance of cladding and structural steels in liquid metal reactors, materials science and technology, in: A Comprehensive Treatment, vol. 10A, VCH Publishers, Weinheim, 1994, pp. 483–514 (Chapter 6).
26. Garner F.A., Radiation-Induced Damage in Austenitic Structural Steels Used in Nuclear Reactors, in: Konings, Rudy JM and Stoller Roger E (eds.) Comprehensive Nuclear Materials 2nd edition, Vol. 3, pp. 57–168. Oxford: Elsevier, 2020.
27. Olander D.R., Fundamental Aspects of Nuclear Reactor Fuel Element, TID 26711-P1, 1976.
28. Heald P.T. and Speight M.V., Point Defect Behaviour in Irradiated Materials, Acta. Metall., Vol. 23, p. 1389, 1975.
29. Griffiths, M.; Ramos-Nervi, J.; Greenwood, L.; A Rate Theory Model of Radiation-Induced Swelling in an Austenitic Stainless Steel. J. Nucl. Eng. 2021, 2, 484-515. https://doi.org/10.3390/jne2040034
30. Heald, P. T. and Speight, M. V., Steady State Irradiation Creep, Philosophical Magazine, Vol. 29, p. 1075, 1974.
31. P.T. Heald and J.E. Harbottle, Irradiation Creep due to dislocation Climb and Glide, J. Nucl. Mater., 67, p. 229-233, 1977.
32. Griffiths M., Effect of Neutron Irradiation on the Mechanical Properties, Swelling and Creep of Austenitic Stainless Steels, Materials 2021, 14(10), 2622; https://doi.org/10.3390/ma14102622
